# Rearrangement Strategy for the Preparation of Polymers With π-Conjugated Structures

**DOI:** 10.3389/fchem.2021.665877

**Published:** 2021-04-01

**Authors:** Jian Tang, Tinghao Xie, Jieting Geng, Jing Hua, Zhaobo Wang

**Affiliations:** Key Laboratory of Rubber-Plastics, Ministry of Education, Shandong Education, Shandong Provincial Key Laboratory of Rubber-plastics, Qingdao University of Science and Technology, Qingdao, China

**Keywords:** conjugated polymers, n-butyllithium, rearrangement, carbanion, polybutadiene

## Abstract

π-Conjugated polymers are usually prepared by polymerization only. In this perspective article, typical synthesis methods of conjugated polymers are briefly summarized, and a novel strategy for preparing conjugated polymers by rearrangement is proposed. During the metalation process, many conjugated structures were generated in polybutadiene by double bond migration. The effects of reaction time, temperature, and catalyst dosage on the product structure were investigated. Moreover, the structure of the products was confirmed by FTIR, ^1^H NMR, and 2D HSQC NMR spectra. Thus, a possible reaction mechanism was proposed, in which polybutadiene generates allylic carbanions in the presence of n-butyllithium, and then the double bonds migrate through the carbanions rearrangement to generate many conjugated structures in the backbone chain. The method shows promise in facile and low-cost synthesis of conjugated polymers without the need for precious metal catalysts.

## Introduction

π-conjugated polymers are polymers containing conjugated structures in the backbone chain. The chemical and physical properties of π-conjugated polymers vary drastically with their structure. The application of these diverse π-conjugated polymers represents a potentially fertile field for chemistry and materials research (Dai et al., 2020; Saito et al., 2020). Recently, there is an increasing interest in conjugated polymers, and their applications are becoming more widespread, such as intrinsically conducting polymers (Aydemir et al., 2016), solar cells (Zhang et al., 2019; Liu et al., 2020), and organic light emitting diodes (OLEDs) (Lozano-Hernández et al., 2020; Milster et al., 2020). Besides, conjugated double bonds have more excellent reactivity compared to isolated double bonds, so conjugated polymers are widely used as substrates to prepare high-performance materials through chemical modification. For instance, conjugated polymers can be modified by cycloaddition reactions to prepare a variety of functional polymers (Yuksekdag et al., 2017), and they can also be used as macromonomers to prepare polymers with complex topologies (Makovetskii et al., 2006; Deepak and Gauthier, 2019), which often have excellent hydrodynamic properties.

Generally, conjugated polymers are prepared mainly by means of polymerization, including coordination polymerization and condensation polymerization. As early as 1958, Natta and co-workers reported the synthesis of polyacetylene using Ti/Al catalysts, which was a method based on coordination polymerization (Saxman et al., 1985). In 2000, Shirakawa et al. awarded the Nobel Prize in Chemistry for their studies in conductive polymers, particularly the doped polyacetylene, thus triggering a boom in the study of conducting polymers based on coordination polymerization (Shirakawa, 2001). In recent years, the application of conjugated polymers in emerging fields such as solar cells and OLEDs has received much attention from researchers. Most of the novel π-conjugated materials used in these fields are synthesized by condensation polymerization, especially Suzuki coupling polymerization (Guo et al., 2013).

The development of novel methods for the synthesis of conjugated polymers has also been a hot topic in this field. Over the years, various palladium- and nickel-catalyzed cross-coupling reactions have been developed, allowing couplings of aryl halides with organometallic aryl derivatives, which provides us with a versatile tool for the preparation of conjugated polymers (Baker et al., 2018). More recently, direct arylation polymerization (DArP) has attracted much attention for its efficiency and environmental friendliness. In DArP, the preparation of monomers is usually facile, no organostannane or organoboron monomers are required, and the by-products are less toxic, in line with the requirements of green chemistry (Bura et al., 2016).

From the above examples, it can be seen that the development of convenient, low-cost and environmentally friendly methods for the synthesis of conjugated polymers is one of the goals pursued by researchers in this field. Besides, the lack of alternative synthesis methods has more or less plagued chemists in the design and preparation of advanced conjugated polymers. Herein, we describe a novel strategy for the preparation of conjugated polymers. We found that the double bonds in polydienes were rearranged in the presence of alkyllithium (Tang et al., 2021). In this work, we have studied this phenomenon in detail and developed a new method for the synthesis of conjugated polymers from non-conjugated (isolated) polymers by a rearrangement strategy. This method is easy to operate, all the substrates are commercially available, and no precious metal catalysts are employed.

## Rearrangement Strategy for Preparing Conjugated Polymers

This work was prompted by the unexpected finding of C=C bond rearrangement and migration of polybutadiene in the presence of n-butyllithium (n-BuLi) and N,N,N′,N′-tetramethylethylenediamine (TMEDA) (Tang et al., 2021). Polybutadiene was dissolved in n-hexane and heated in the presence of butyllithium/TMEDA to prepare samples for analysis. Details of the experimental procedure can be found in the Supplementary Material. The products of rearrangement of polybutadiene were analyzed by FTIR and NMR spectroscopy.

**Figure 1:**
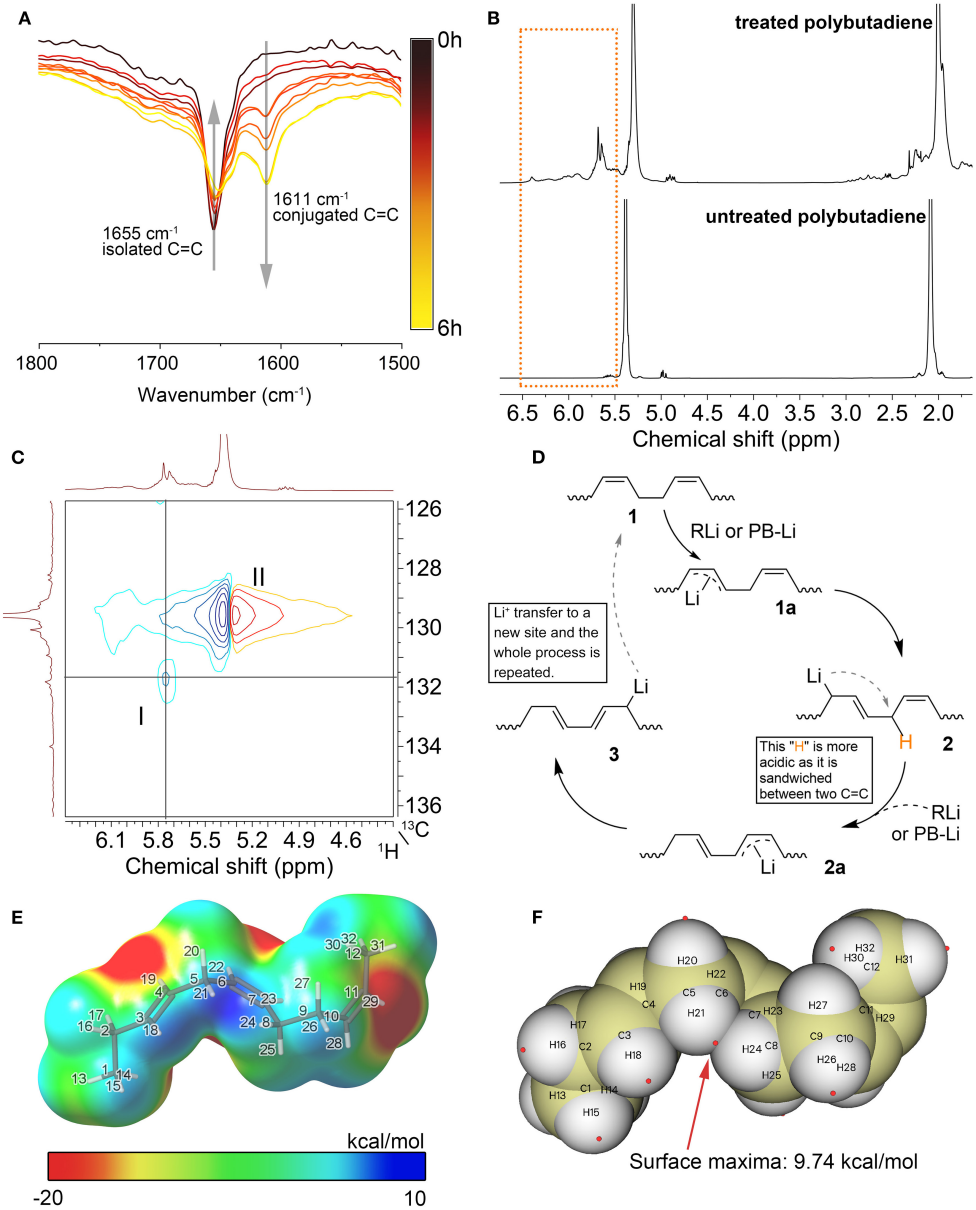
**(A)** FTIR spectra of treated polybutadiene with different reaction times. **(B)** ^1^H NMR spectra for PB and its rearrangement product. Reaction condition: 1 h, 80°C, in n-hexane, TMEDA/n-BuLi = 1:1, the molar ratio of n-BuLi to C=C is 1:1. **(C)** 2D ^1^H-^13^C HSQC NMR of treated polybutadiene. The reaction condition is the same as before. **(D)** Proposed reaction mechanism. **(E)** ESP mapped van der Waals surface for the model of key intermediate. **(F)** Distribution of ESP maxima (red spheres) on the van der Waals surface of the model. Preparation and analysis procedures for samples in this figure are given in Supplementary Material.

Spectroscopic studies of the products confirm the presence of a large number of conjugated double bonds in the n-BuLi-treated polybutadiene. In the FTIR spectrum of the treated polybutadiene (Figure 1A), The peak at 1,655 cm^−1^ assigned to the C=C stretching vibration gradually shifted to 1,611 cm^−1^ as the reaction time increases. Such a significant redshift is attributed to the averaging of the electron density due to the formation of the conjugated double bonds. In addition, the broad signal at 5.7 ppm in the ^1^H NMR spectrum of the treated polybutadiene in Figure 1B is assigned to protons in the conjugated structures, which shifted to lower fields compared to protons in the unconjugated structure (5.4 ppm). The stacking of multiple conjugated structures leads to the further dispersion of the electron cloud, which can explain the weak signals appearing at lower fields (5.8–6.5 ppm). The continuous conjugated structures obtained by rearrangement are similar to that of polyacetylene. These signals in the low field are consistent with the pioneers' reports about NMR spectra of polyacetylene (Buskuhl et al., 2009). Moreover, the 2D HSQC spectrum shown in Figure 1C confirms the plausibility of the above assignment. The cross-correlation peak I indicates that the signal at 5.7 ppm (^1^H) is highly correlated with the signal at 131.8 ppm (^13^C), and both the signals at 131.8 ppm in the ^13^C NMR spectrum and 5.7 ppm in the ^1^H NMR spectrum are from carbons or protons of the conjugated structures.

From the above FTIR spectra as well as NMR spectral evidence, it can be deduced that conjugated double bond appeared in polybutadiene after treated with n-BuLi/TMEDA. Thus, we propose a possible reaction mechanism based on the characterization of the product structure. As shown in Figure 1D, the first step of the process is called the lithium-hydrogen exchange reaction or lithiation reaction. The n-BuLi abstracts a proton from polybutadiene and then forms the allylic carbanion intermediate **1a**. Next, the carbanion **1a** undergoes a rearrangement process to form intermediate **2**. As we can see, the proton sandwiched between the two C=C bonds in intermediate **2** is more acidic because the two double bonds disperse the electron density. This speculation was supported by density functional theory (DFT) calculations. Considering that the polymer contains too many atoms, making it difficult to calculate, we designed an oligomer containing only a few structural units as a model, thus simplifying the calculation. The DFT calculations were implemented by ORCA 4.2.1 program package (Neese, 2011). The electrostatic potential (ESP) analysis was performed with the Multiwfn 3.7 program (Lu and Chen, 2012). Figure 1E shows the ESP colored van der Waals surface for the model of intermediate 2. The blue region in this figure has a positive ESP, which is more likely to be attacked by a negatively charged Lewis base such as n-BuLi (Liu et al., 2021). As shown in this figure, the maximum electrostatic potential energy is found near the region sandwiched by two C=C bonds (Maximum value = 9.74 kcal/mol, all extreme values and their distribution are given in the Supplementary Material). Therefore, the protons in this area are more likely to be abstracted by alkyllithium, forming a new allylic carbanion **2a**. As with the transition process from **1a** to **2**, intermediate **2a** tends to resonate into the more stable structure **3** because the conjugated structure has the lowest energy. This process is repeated throughout the polymer chain until most of the isolated double bonds are converted into conjugated double bonds. Notably, n-BuLi is not the only lithium species that can extract protons from the polymer. The lithiated polybutadiene (PB-Li) can also work as a Lewis base to extract protons from the polymer chain. This process is quite like a chain transfer process, where lithium atoms are transferred from a site to another so that lithium can be recycled in this process.

Moreover, a series of rearrangement reactions were carried out at different reaction temperatures, times, and catalyst dosages to optimize the reaction conditions. As shown in entries 1–9 in Table 1, the proportion of conjugated double bonds to the total double bonds in the products increased with increasing reaction time. The reaction is a relatively slow process, so the content of conjugated double bonds in the product can be regulated by controlling the reaction time. As with most reactions, the dosage of catalyst directly affects the conversion. When the dosage of n-BuLi was 10% double bond equivalent, the percentage of conjugated double bonds in the product was only 9.6% (The molar ratio of conjugate double bonds to all double bonds, same below) at 60 min of reaction, while the percentage of conjugated double bonds in the product was 16.9, 23.9, and 40.2% when the dosage of n-BuLi was 30, 50, and 100% double bond equivalent. Besides, the temperature also strongly affected the reaction. Almost no conjugated double bonds were detected in the products when the reaction was carried out at 0°C for 60 min. At a reaction temperature of 30°C, the percentage of conjugated double bonds in the resulting polymers was only 5.6%, whereas approaching 20.0% at 60°C. Consequently, we can regulate the amount of conjugated double bonds freely by controlling the conditions such as temperature, reaction time, and catalyst dosage.

**Table 1:** Optimization of reaction conditions^a^.

**Entry**	**Time**	**T**	**[Li]/[PB]^**b**^**	**C. content^**c**^**
1	0 min	/	/	0%
2	5 min	80°C	1	11.9%
3	15 min	80°C	1	31.4%
4	30 min	80°C	1	35.2%
5	60 min	80°C	1	40.2%
6	90 min	80°C	1	43.9%
7	120 min	80°C	1	44.8%
8	240 min	80°C	1	56.8%
9	360 min	80°C	1	68.9%
10	60 min	80°C	0.1	9.6%
11	60 min	80°C	0.3	16.9%
12	60 min	80°C	0.5	23.9%
13	60 min	0°C	1	trace
14	60 min	30°C	1	5.6%
15	60 min	60°C	1	20.0%

a *Reaction condition: in n-hexane, TMEDA/n-BuLi = 1:1. The samples preparation procedures are given in the Supplementary Material*.

b *The molar ratio of n-BuLi to C=C bond*.

c *C. content: conjugated double bond content, i.e., the molar proportion of conjugated C=C bonds to all C=C bonds. It was determined by ^1^H NMR*.

## Conclusions

The n-BuLi/TMEDA system can induce double bond migration in polybutadiene, which results in the generation of π-conjugated structures. The kinetic study of the reaction shows that the rearrangement process occurs gradually, so the content of conjugated double bonds in the products can be regulated by controlling temperature, reaction time, and catalyst dosage. Furthermore, based on spectroscopic evidence, the reaction mechanism was described as a process in which carbanion rearrangement leads to the conversion of the double bond to a more stable conjugated structure.

Since the resulting polymer does not contain aromatic rings, it is not suitable as a conductive polymer or for making optoelectronic devices. However, the conjugated double bonds give it excellent reactivity, so it can be used as a substrate to prepare various functional materials by chemical modification. For example, it can be used as a backbone to prepare comb polymer by grafting polymerization. It can also be chemically modified to prepare functional polymer materials with various functional groups. We have to admit that this strategy has some shortcomings yet, such as narrow substrate scope and low stereoselectivity, which may limit its application at this stage. However, this method does not involve precious metal catalysts such as palladium, and all substrates are low-cost commercially available reagents, which is in line with the concept of sustainable development. This idea of preparing conjugated polymers from ready-made polymers is promising, and we hope that this work provides new ideas for the research of π-conjugated polymer synthesis.

## Data Availability Statement

The original contributions generated for the study are included in the article/Supplementary Material, further inquiries can be directed to the corresponding author/s.

## Author Contributions

JT and TX prepared materials and carried out experiments. JG helped to characterize materials. JH and ZW supervised the work. All authors contributed to revising the manuscript and approved the final version.

## Conflict of Interest

The authors declare that the research was conducted in the absence of any commercial or financial relationships that could be construed as a potential conflict of interest.
