# Supplementary material for: Rearrangement Strategy for the Preparation of Polymers With π-Conjugated Structures
Source: Front Chem. 2021 Apr 1;9:665877. doi: 10.3389/fchem.2021.665877 (PMC8049560; doi:10.3389/fchem.2021.665877)
Supplement: Supplementary file 2 [file Data_Sheet_1.PDF]

# *Supplementary Material*

## 1 Cartesian Coordinates of Optimized Structure

**Table S1.** Cartesian coordinates for the optimized geometry of the model

| Atom Symbol | X | Y | Z |
|---|---|---|---|
| C | -5.18188 | 1.32159 | 0.595059 |
| C | -4.89703 | -0.07876 | 0.026858 |
| C | -3.49579 | -0.22076 | -0.502901 |
| C | -2.58558 | -1.0898 | -0.058304 |
| C | -1.17678 | -1.22427 | -0.58746 |
| C | -0.13823 | -1.05116 | 0.497343 |
| C | 0.83603 | -0.137102 | 0.571812 |
| C | 1.165515 | 0.949751 | -0.414995 |
| C | 2.627394 | 0.883439 | -0.92918 |
| C | 3.647719 | 1.158801 | 0.142978 |
| C | 4.623448 | 0.356181 | 0.585233 |
| C | 4.956533 | -1.03449 | 0.123596 |
| H | -6.21678 | 1.403587 | 0.943185 |
| H | -4.51957 | 1.543607 | 1.437726 |
| H | -5.02327 | 2.095731 | -0.16397 |
| H | -5.61492 | -0.28302 | -0.78091 |
| H | -5.07665 | -0.83661 | 0.799009 |
| H | -3.22045 | 0.462007 | -1.30993 |
| H | -2.85576 | -1.766399 | 0.754782 |
| H | -1.05708 | -2.230897 | -1.02007 |
| H | -1.02244 | -0.515152 | -1.40604 |
| H | -0.22046 | -1.76509 | 1.317863 |
| H | 1.492983 | -0.17371 | 1.440428 |
| H | 0.487233 | 0.922519 | -1.27318 |
| H | 1.014635 | 1.928269 | 0.065446 |
| H | 2.739279 | 1.637666 | -1.72133 |
| H | 2.798276 | -0.089781 | -1.39938 |
| H | 3.560915 | 2.142664 | 0.607177 |

## 2 Distribution of ESP Maxima (red spheres) on the Van Der Waals Surfaces of the Model

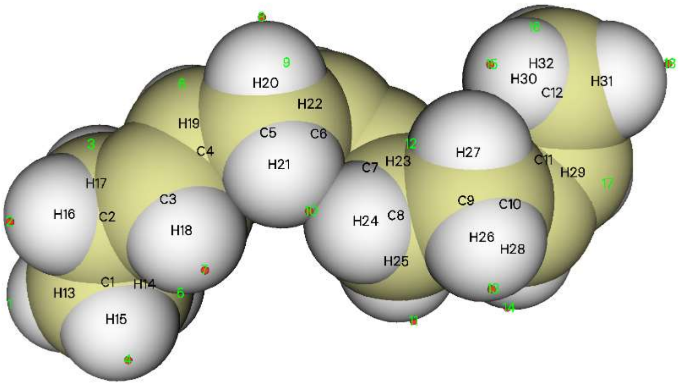


Number of surface minima: 7

| # | a.u. | eV | kcal/mol | X/Y/Z coordinate (Angstrom) | | |
|---|---|---|---|---|---|---|
| 1 | -0.00386895 | -0.105279 | -2.427802 | -5.599888 | 2.750863 | 1.875348 |
| 2 | -0.02356154 | -0.641142 | -14.785105 | -3.774043 | -2.024568 | -1.576814 |
| 3 | -0.03072818 | -0.836156 | -19.282242 | -2.059200 | 0.577141 | 1.028860 |
| * 4 | -0.03145928 | -0.856051 | -19.741012 | -1.112889 | 0.558201 | 1.365423 |
| 5 | -0.02396732 | -0.652184 | -15.039735 | 1.721677 | -1.965508 | -0.058551 |
| 6 | -0.01994384 | -0.542699 | -12.514958 | 3.252824 | 0.385645 | 2.184191 |
| 7 | -0.02436794 | -0.663085 | -15.291124 | 5.448389 | 1.628910 | -0.908702 |

Number of surface maxima: 18

| # | a.u. | eV | kcal/mol | X/Y/Z coordinate (Angstrom) | | |
|---|---|---|---|---|---|---|
| 1 | 0.01054812 | 0.287029 | 6.619051 | -7.536202 | 1.447866 | 1.340301 |
| 2 | 0.00979239 | 0.266464 | 6.144820 | -6.597212 | -0.331742 | -1.762724 |
| 3 | 0.01032303 | 0.280904 | 6.477802 | -5.239183 | -1.757305 | 1.823629 |
| 4 | 0.01099696 | 0.299243 | 6.900705 | -4.807896 | 2.866746 | -1.288732 |
| 5 | 0.00632129 | 0.172011 | 3.966671 | -3.918716 | 1.935543 | 2.611293 |
| 6 | 0.01306024 | 0.355387 | 8.195432 | -2.987151 | -2.730894 | 1.737184 |
| 7 | 0.01339903 | 0.364606 | 8.408024 | -2.843003 | 1.284206 | -2.359027 |
| 8 | 0.01069911 | 0.291137 | 6.713796 | -0.889047 | -3.538474 | -1.449684 |
| 9 | 0.01191443 | 0.324208 | 7.476425 | -0.586616 | -2.818409 | 2.139161 |
| * 10 | 0.01551499 | 0.422184 | 9.735812 | -0.613786 | 0.491698 | -2.426610 |
| 11 | 0.01050048 | 0.285733 | 6.589156 | 1.149866 | 3.270632 | 0.415086 |
| 12 | 0.00566381 | 0.154120 | 3.554094 | 1.854102 | -0.535006 | 2.719760 |
| 13 | 0.01151400 | 0.313312 | 7.225149 | 2.643038 | 2.608407 | -2.709137 |
| 14 | 0.01236273 | 0.336407 | 7.757739 | 3.261570 | 3.408155 | 1.082208 |
| 15 | 0.01338772 | 0.364298 | 8.400929 | 3.489109 | -1.767975 | -1.755788 |
| 16 | 0.01233203 | 0.335572 | 7.738472 | 5.020975 | -2.635040 | 2.001773 |
| 17 | 0.01257534 | 0.342192 | 7.891153 | 6.113265 | 1.139600 | 2.389102 |

| | | | | | | |
|---|---|---|---|---|---|---|
| 18 | 0.01248122 | 0.339631 | 7.832091 | 7.262942 | -1.195586 | -0.746107 |

# 3 NMR & FTIR Spectra

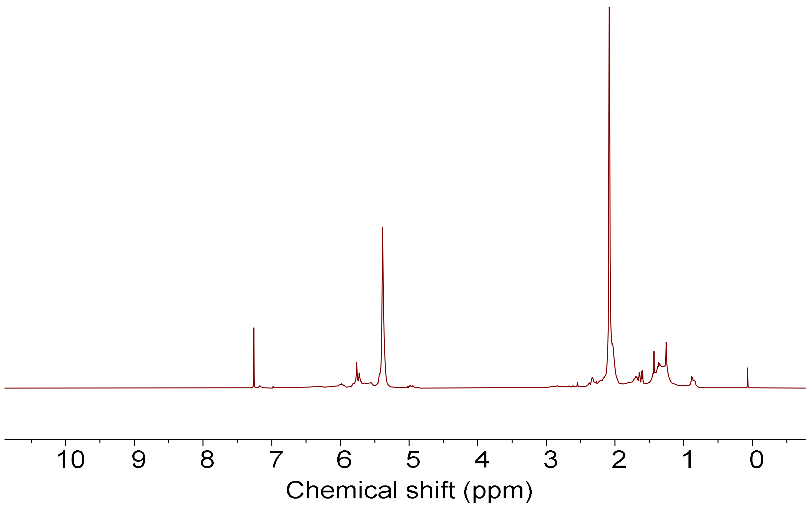


**Figure S1.** $^{1}H$ NMR spectrum of rearrangement product (Reaction condition: 1h, 80°C, in n-hexane, TMEDA/n-BuLi=1:1, the molar ratio of n-BuLi to C=C is 1:1.).

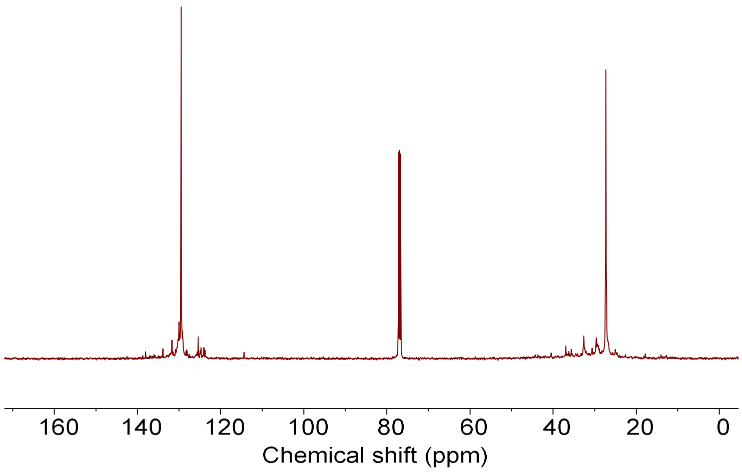


**Figure S2.** $^{13}C$ NMR spectrum of rearrangement product (Reaction condition: 1h, 80°C, in n-hexane, TMEDA/n-BuLi=1:1, the molar ratio of n-BuLi to C=C is 1:1).

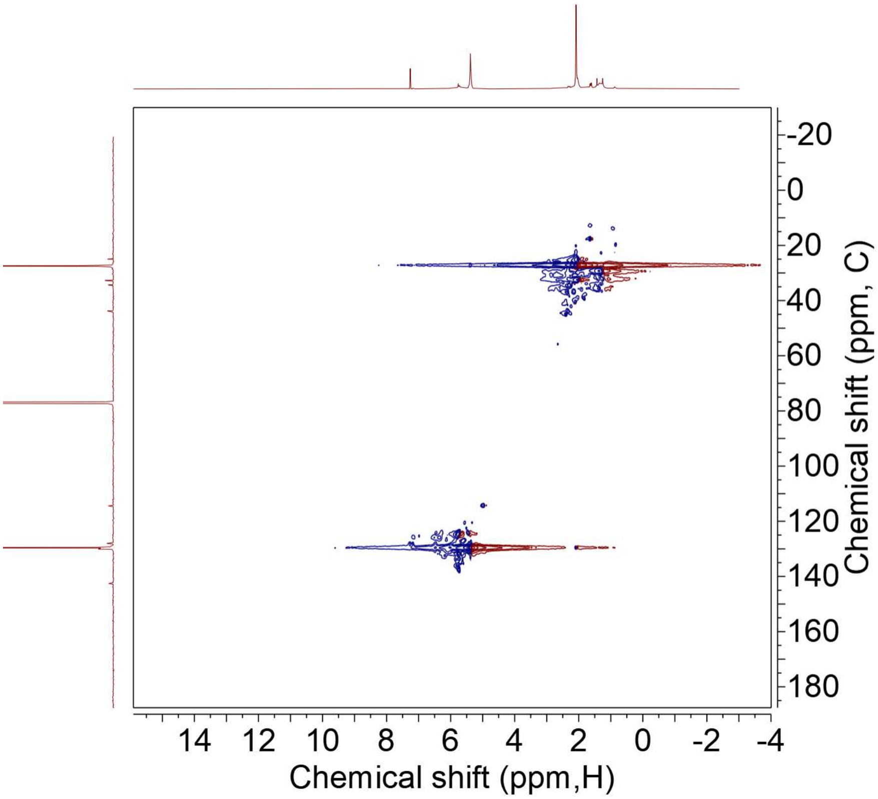


**Figure S3.** 2D HSQC NMR of treated sample.

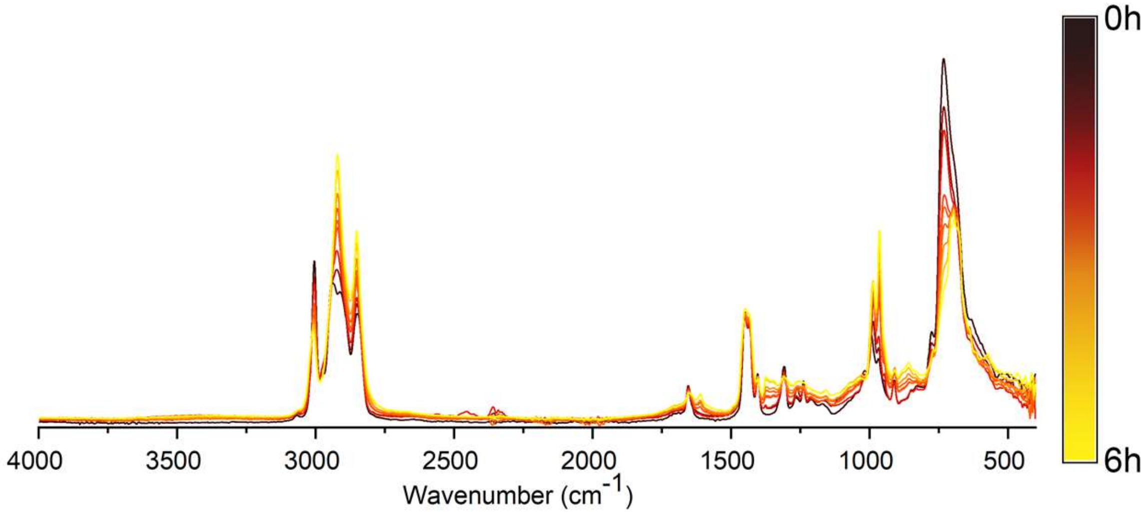


**Figure S4.** FTIR spectrum of rearrangement product (Reaction condition: 80°C, in n-hexane, TMEDA/n-BuLi=1:1, the molar ratio of n-BuLi to C=C is 1:1).

## 4 Preparation and Analysis Procedures for Samples in Figure 1 and Table 1

**Materials & Characterizations.** TMEDA was purchased from Sigma-Aldrich Chemical Co. and freshly distilled in the presence of CaH. n-Hexane was distilled in the presence of Na/K and benzophenone under a dry $N_2$ atmosphere. n-Butyllithium (2.5 mol/L in hexane), methanol, and 2,6-di-tert-butyl-4-methylphenol were obtained from Energy Chemical and used without further purification. Polybutadiene (Grade: BR9000. 1,4-cis > 97%, determined by FTIR spectroscopy.) was supplied from Sinopec Qilu Petrochemical Company and used without further purification. $^1$H NMR and 2D HSQC NMR were measured on a Bruker Avance 500M spectrometer in $CDCl_3$ at 30°C. FTIR spectra were recorded on a Bruker Tensor II FTIR spectrometer. Conjugated double bond content was quantitatively determined by $^1$H NMR integration, the equation employed for the calculation is shown as follows.

$$C.content = \frac{I(5.74\sim6.62)}{I(5.74\sim6.62) + I(5.4) + I(4.99)} \times 100\%$$

**Sample preparation.** The typical sample preparation procedure was as follows: A certain amount of polybutadiene/n-hexane solution (0.067 g/mL), TMEDA, and n-BuLi were added sequentially into a glass reactor under a nitrogen atmosphere. After heating in a water bath at a given temperature for a given time, methanol containing 2,6-di-tert-butyl-4-methylphenol (1.0 wt%) was added to quench the reaction. The reaction product was washed several times with 10% dilute hydrochloric acid and water to remove the lithium salt and remaining TMEDA. The organic layer was separated and dried with $MgSO_4$, then the suspension was filtered, and the filtrate was evaporated under vacuum.

**Computational details.** Considering that the polymer contains too many atoms, making it difficult to calculate, we designed an oligomer containing only a few structural units as a model, thus simplifying the calculation. The DFT calculations were implemented by ORCA 4.2.1 program package (Neese 2011). The equilibrium geometry of the model was optimized by using DFT methods at the B3LYP/6-31G(d,p) level (Lee, Yang et al. 1988). The frequency calculation was performed to characterize the minimum structure as a stable point on the potential energy surface (no imaginary frequency). The ESP analysis was performed with the Multiwfn 3.7 program (Lu and Chen 2012). The visualization of the ESP map was performed with Avogadro 0.9.5 (Hanwell, Curtis et al. 2012) and Jmol 14.31.27 (Howard 2013). Besides, POV-Ray software was employed for 3D rendering to obtain higher resolution images.

**References**

Hanwell, M. D., D. E. Curtis, D. C. Lonie, T. Vandermeersch, E. Zurek and G. R. Hutchison (2012). "Avogadro: an advanced semantic chemical editor, visualization, and analysis platform." J Cheminform **4**(1): 17.

Howard, M. (2013). Jmol: an open-source Java viewer for chemical structures in 3D.

Lee, C., W. Yang and R. G. Parr (1988). "Development of the Colle-Salvetti correlation-energy formula into a functional of the electron density." Phys Rev B Condens Matter **37**(2): 785-789.

Lu, T. and F. Chen (2012). "Multiwfn: a multifunctional wavefunction analyzer." J Comput Chem **33**(5): 580-592.

Neese, F. (2011). "The ORCA program system." WIREs Computational Molecular Science **2**(1): 73-78.
